# Andrographolide inhibits the activation of NF-κB and MMP-9 activity in H3255 lung cancer cells

**DOI:** 10.3892/etm.2013.1196

**Published:** 2013-07-02

**Authors:** WEIMIN LUO, YUEFENG LIU, JUN ZHANG, XIANGYU LUO, CHENYI LIN, JIALONG GUO

**Affiliations:** 1 Department of Cardiothoracic Surgery, Taihe Hospital Affiliated to Hubei University of Medicine, Shiyan, Hubei 442000, P.R. China; 2 Department of Ophthalmology, People’s Hospital Affiliated to Hubei University of Medicine, Shiyan, Hubei 442000, P.R. China

**Keywords:** andrographolide, lung cancer cells, NF-κB, MMP-9

## Abstract

This study aimed to determine the effect of andrographolide (AD) on the growth of H3255 lung cancer cells and its possible impact on the expression and activity of the matrix metalloproteinase (MMP)-9 protein. H3255 cells were cultured *in vitro*, and treated with AD (1, 5 or 10 μM) for 24, 48 or 72 h. Cell proliferation was detected using an MTT assay and the expression of MMP-9 mRNA was measured using a reverse transcription-polymerase chain reaction (RT-PCR). The activity of MMP-9 was assessed by gelatin zymography, while the nuclear translocation of the nuclear factor-κB (NF-κB) p65 subunit and the phosphorylation of IκB were determined by western blotting. AD inhibited the proliferation of the H3255 cells in a concentration- and time-dependent manner, in addition to downregulating the expression of MMP-9 mRNA and the activity of MMP-9. Moreover, AD significantly inhibited the nuclear translocation of the NF-κB p65 subunit and suppressed IκB phosphorylation. The significant inhibition of H3255 cell proliferation by AD may have been correlated with the reduction in MMP-9 expression and activity through the inhibition of the phosphorylation of IκB and the translocation of NF-κB. The results suggest that AD is a promising drug candidate for the treatment of the migration and invasion of malignant tumors.

## Introduction

Metastasis and invasiveness are two of the most significant characteristics of malignant tumor cells. Among the proteins involved in metastasis and invasiveness, matrix metalloproteinase-9 (MMP-9), a member of the MMP family, is particularly important, due to its ability to degrade type IV collagen fibers and the extracellular matrix (1). Studies have demonstrated that the promoter region of MMP-9 contains cis-acting elements and binding loci for the transcription factors nuclear factor-κB (NF-κB) and activator protein AP-1. Cytokines and phorbol 12-myristate 13-acetate (PMA) are able to stimulate the production of MMP-9 by activating NF-κB and AP-1, which indicates that the expression of MMP-9 is inducible (2). As a result, proteins regulating the expression and activity of MMP-9 are promising drug targets for antitumor studies (3).

Numerous extracts of herbal medicines have been demonstrated to exhibit antitumor activities, including the inhibition of tumor cell growth and metastasis. Andrographolide (AD) is a type of diterpenoid extracted from the medicinal plant *Andrographis paniculata*, which is usually used to treat infectious diseases (4). It has been observed that AD inhibits the proliferation of multiple types of tumor cells, including leukemia, glioma, prostatic carcinoma and breast cancer cells (5,6). However, its effect in the treatment of lung cancer is unknown. The aim of the present study was to investigate how AD affected PMA-induced MMP-9 expression, in addition to studying the potential regulatory molecules and the mechanisms involved.

## Materials and methods

### Reagents

AD and gelatin were purchased from Sigma-Aldrich (St. Louis, MO, USA) and PMA was obtained from Calbiochem (La Jolla, CA, USA). Anti-p65, β-actin and anti-IκB antibodies were purchased from Santa Cruz Biotechnology, Inc., (Santa Cruz, CA, USA), while anti-phospho-IκB antibody was purchased from Cell Signaling Technology, Inc. (Beverly, MA, USA). Any additional analytical reagents were purchased from Sangon Biotech (Shanghai) Co., Ltd. (Shanghai, China) and Amerco (Reno, NV, USA).

### Cell culture

Non-small-cell H3255 lung cancer cells were purchased from ATCC (Manassas, VA, USA) and cultured in RPMI-1640 medium (pH 7.4) containing 10% fetal bovine serum (FBS), 100 U/ml penicillin and 100 U/ml streptomycin at 37°C with 5% CO_2_. Cell density was adjusted to 1×10^5^ cells/ml prior to the tests. The cells were divided into several AD-treated groups, which were treated with 1, 5 or 10 μM AD at 37°C for 24, 48 or 72 h. The negative control groups were treated with equal volumes of RPMI-1640 medium, containing dimethylsulfoxide (DMSO) at a final concentration of 0.1%.

### MTT assay

Cells were inoculated onto 96-well plates at 1×10^4^ cells per well, treated with PMA for 24 or 48 h and then supplemented with 1.0, 5.0 and 10.0 μM of AD. Following processing, the supernatant was disposed of and DMSO solution containing MTT was added. The absorbance values were determined at 550 nm.

### RT-PCR

Total RNA was extracted using TRIzol^®^ reagent (Invitrogen Life Technologies, Carlsbad, CA, USA) and 2 μg total RNA was used to prepare the cDNA with a SuperScript^®^ First Strand cDNA Synthesis System kit (Invitrogen Life Technologies). The PCR products were stained with ethidium bromide, following electrophoresis in 2% agarose gel. The primers used for MMP-9 were 5′-TCCCTGGAGACCTGA GAACC-3′ and 5′-CGGCAAGTCTTCCGAGTAGTT-3′, while the primers used for glyceraldehyde 3-phosphate dehydrogenase (GAPDH) were 5′-CCATCACCATCTTCCAGGAG-3′ and 5′-CCTGCTTCACCACGTTCTTG-3′.

### Gelatin zymography

The cells were cultured in serum-free medium for 24 h. Following this, the supernatant was collected, supplemented with Laemmli sample buffer and subjected to sodium dodecyl sulfate-polyacrylamide gel electrophoresis (SDS-PAGE) with 1 mg/ml gelatin (separation gel 10%). Subsequent to the electrophoresis, the gel was kept in renaturation buffer containing 2.5% Triton-X-100 for 30 min, in order to remove the SDS, and then incubated in a buffer containing 50 mM HCl (pH 7.4), 5 mM CaCl_2_ and 1 μM ZnCl_2_ at 37°C overnight. The gel was subsequently stained with 0.05% Coomassie Brilliant Blue R-250 at room temperature for 30 min, prior to being decolorized with deionized water for photography and grey-scale scanning.

### Enzyme-linked immunosorbent assay (ELISA)

The supernatant of the cell culture was collected and the MMP-9 activity was tested with a SensoLyte Plus™ 520 MMP-9 assay kit (AnaSpec, Inc., Fremont, CA, USA). The unit of enzymatic activity was indicated as 490 nm (excitation wavelength)/520 nm (emission wavelength).

### Immunoblotting assay

The nuclear and cytoplasmic proteins were extracted in accordance the protocol provided with the ProteoJET™ Cytoplasmic and Nuclear Protein Extraction kit (Fermentas, Vilnius, Lithuania). Proliferating cell nuclear antigen (PCNA) and α-tubulin were used as internal controls for nuclear and cytoplasmic proteins, respectively. The isolated proteins were subjected to SDS-PAGE and transferred onto nitrocellulose membranes, prior to being blocked in Tris-buffered saline (TBS) containing 0.1% Tween-20 and 5% non-fat dry milk. The proteins were then incubated and treated with primary and secondary antibodies for enhanced chemiluminescence (ECL) detection.

### Statistical analyses

The data were analyzed with the statistical analysis software SPSS 15.0 (SPSS, Inc., Chicago, IL, USA). The values are presented as the mean ± standard deviation. One-way analysis of variance (ANOVA) was used for multi-group comparisons with a Student’s t-test. P<0.05 was considered to indicate a statistically significant difference.

## Results

### AD inhibits the proliferation of H3255 cells

To investigate whether AD affected the proliferation of H3255 cells, the cells were treated with 100 nM PMA in the absence or presence of AD (1, 5 or 10 μM) for 24, 48, or 72 h. The negative control cells were treated with equal volumes of RPMI-1640 medium containing DMSO (0.1%) only. As shown in Table I, AD significantly inhibited the proliferation of H3255 cells *in vitro*. The inhibition rates of the H3255 cells increased when the concentration of AD was increased and when the treatment duration was longer. At the concentrations used in the study, AD did not appear to exert any toxic effects on the cells. These results demonstrate that AD inhibited the proliferation of H3255 cells in a concentration- and time-dependent manner.

### AD inhibits the PMA-induced expression of MMP-9

**Figure 1:**
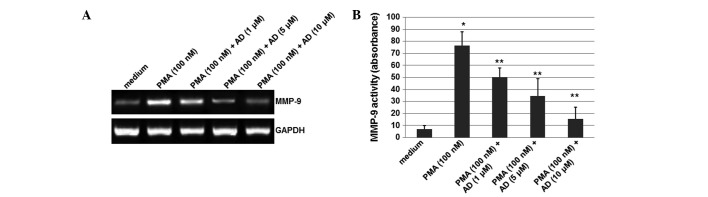
Effect of andrographolide (AD) on the expression and activity of the phorbol 12-myristate 13-acetate (PMA)-induced matrix metalloproteinase (MMP)-9 mRNA and protein in H3255 cells. (A) H3255 cells were incubated with the indicated concentrations of AD for 30 min, prior to stimulation with 100 nM PMA for 24 h. The mRNA level of endogenous MMP-9 was measured by a reverse transcription-polymerase chain reaction (RT-PCR) and glyceraldehyde 3-phosphate dehydrogenase (GAPDH) was used as an internal control. (B) H3255 cells were pretreated with AD for 30 min and stimulated with 100 nM PMA for 24 h. After 24 h, the conditioned medium was collected and assayed for the quantity and the activity of secreted MMP-9 using gelatin zymography. ^*^P<0.05 vs. the control group (medium). ^**^P<0.01 vs. the PMA only group.

To detect the effect of AD on MMP-9, the total RNA was extracted from the cells treated with or without AD, and RT-PCR was performed. As shown in Fig. 1A, although the mRNA levels of MMP-9 were significantly increased by PMA, this induction of MMP-9 expression was reduced by treatment with AD (1, 5 or 10 μM) in a concentration-dependent manner. In this experiment, GAPDH was used as an internal control. These results suggest that AD inhibited the PMA-induced expression of MMP-9.

### AD inhibits the MMP-9 activity induced by PMA

To detect whether the MMP-9 activity was affected by AD, a gelatin zymography experiment was performed. As shown Fig. 1B, although the activity of MMP-9 increased upon treatment with PMA, AD (1, 5 or 10 μM) decreased this activity in a concentration-dependent manner. The MMP-9 activity was reduced by 44% when the cells were treated with 10 μM AD. These results indicate that AD inhibits the MMP-9 activity induced by PMA.

### AD inhibits the PMA-induced translocation of the NF-κB p65 subunit and the IκB phosphorylation

**Figure 2:**
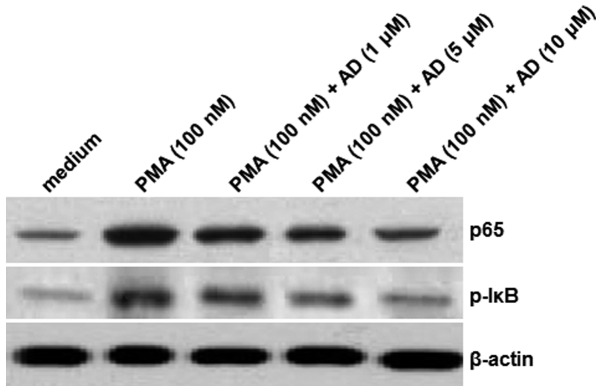
Effect of andrographolide (AD) on the activity of nuclear factor-κB (NF-κB). H3255 cells were pretreated with the indicated amounts of AD for 30 min and then stimulated with 100 nM phorbol 12-myristate 13-acetate (PMA) for 30 min. Cells were harvested and fractionated into the cytoplasm and the nucleus. The lysates were then separated on a 10% sodium dodecyl sulfate-polyacrylamide gel and subjected to western blotting with anti-p65, -p-IκB and -IκB antibodies. The analysis was repeated three times. β-actin was used as a control. p-IκB, phosphorylated IκB.

To further investigate the mechanisms of the effects exerted by AD, the protein levels of the NF-κB p65 subunit were determined using an immunoblotting assay. As shown in Fig. 2, there was an increase in the level of the NF-κB p65 protein subunit in the nucleus following the induction by PMA. However, the levels of p65 protein in the nucleus were reduced following the treatments with increasing concentrations of AD. Furthermore, although the IκB phosphorylation was significantly increased by PMA, AD attenuated this increase in a concentration-dependent manner. In this experiment, β-actin served as the internal control. These results suggest that AD inhibits the PMA-induced increase in the levels of the NF-κB p65 subunit and IκB phosphorylation.

## Discussion

A number of chemical compounds have been demonstrated to exert antitumor effects on various tumors, including leukemia and prostatic, breast and pancreatic cancer. AD has been revealed to reduce the activity of Na^+^/K^+^-ATPase and inhibit the translocation of NF-κB through regulating proteases (7). It has been indicated that AD exhibits certain inhibitory effects on metastasis in lung cancers (8). However, no comprehensive studies in this field have been performed, and, therefore, further investigations are required to study the exact molecular mechanisms of the antitumor effect of AD.

In the present study, the effect of AD on PMA-induced H3255 cell proliferation was investigated, and it was demonstrated that (i) AD is able to inhibit the expression and activity of MMP-9 and (ii) AD is capable of inhibiting NF-κB-mediated MMP-9 expression by suppressing the activation of NF-κB, and thus preventing the migration and invasion of tumor cells.

Metastasis is a multi-stage complex process involving cell proliferation and migration into the circulatory system, and tumor growth at the primary site. The expression of MMP-9 in numerous types of cancer is increasingly gaining focus, and PMA is a commonly used chemical inducer of tumors *in vivo* and *in vitro*(9). Studies have revealed that PMA is able to enhance the migration and invasion of tumor cells by inducing the expression of MMPs-2 and -9 in glioma, in addition to colon, liver and breast cancer (10), although the mechanisms involved in the PMA-induced invasion in lung cancer have not yet been elucidated. The present study demonstrated that PMA enhanced the expression of MMP-9 in H3255 cells at the mRNA and protein levels, and that AD was capable of inhibiting this effect. The inhibitory effect of AD on the enzymatic activity and protein expression of MMP-9 indicated that AD participates in the regulation of posttranscriptional pathways.

The promoter region of MMP-9 contains regulatory elements for NF-κB and AP-1 (11), making the 5′ regulatory region of MMP-9 gene highly inducible. In order to clarify the relevant mechanisms, we focused on the nuclear translocation of the NF-κB p65 subunit and the phosphorylation of IκB, which have been demonstrated to be necessary for the PMA induction of MMP-9, and which are suppressible using AD. Previous studies have indicated that NF-κB is important in the PMA-induced expression of MMP-9 in lung cancer (12). The results of the present study demonstrated that the NF-κB pathway also facilitates the inhibition of the PMA-induced expression of MMP-9 by AD.

In conclusion, the present study demonstrated that AD is able to inhibit the migration and invasion of lung cancer cells by suppressing the PMA-induced expression of MMP-9, the mechanisms of which may involve the suppression of IκB phosphorylation and the consequent NF-κB activation. It is thus suggested that AD may a promising drug candidate for the clinical treatment of the migration and invasion of malignant tumor cells.

## Figures and Tables

**Table I:** Inhibitory effect of AD on the PMA-induced proliferation of H3255 cells.

	Duration of treatment		
	
AD concentration (μM)	24 h	48 h	72 h
0.0	0.00±0.00	0.00±0.00	0.00±0.00
1.0	15.34±2.38^a^	26.84±1.41^a^^b^	35.23±1.01^a^^b^
5.0	29.28±2.07^a^	34.25±2.51^a^^b^	46.22±1.82^a^^b^
10.0	41.91±1.75^a^	48.51±2.31^a^^b^	59.07±1.43^a^^b^

Values are presented as the mean ± standard deviation.

a P<0.05, compared with the respective 0 μM AD group;

b P<0.05, compared with the inhibitory effect in the respective 24 h group.

AD, andrographolide; PMA, phorbol 12-myristate 13-acetate.
